# Supplementary material for: Iterative improvement in the automatic modular design of robot swarms
Source: PeerJ Comput Sci. 2020 Dec 7;6:e322. doi: 10.7717/peerj-cs.322 (PMC7924708; doi:10.7717/peerj-cs.322)
Supplement: Supplemental Information 3 [file peerj-cs-06-322-s003.zip › argos3/doc/api/standalone/a00494_source.html]

ARGoS: plugins/robots/generic/control\_interface/ci\_differential\_steering\_sensor.cpp Source File


- Main Page
- Related Pages
- Namespaces
- Classes
- Files

- File List
- File Members

# plugins/robots/generic/control\_interface/ci\_differential\_steering\_sensor.cpp

Go to the documentation of this file.

```
00001 
00007 #include "ci_differential_steering_sensor.h"
00008 
00009 #ifdef ARGOS_WITH_LUA
00010 #include <argos3/core/wrappers/lua/lua_utility.h>
00011 #endif
00012 
00013 namespace argos {
00014    
00015   /****************************************/
00016   /****************************************/
00017    
00018 #ifdef ARGOS_WITH_LUA
00019   void CCI_DifferentialSteeringSensor::CreateLuaState(lua_State* pt_lua_state) {
00020     CLuaUtility::OpenRobotStateTable(pt_lua_state, "wheels");
00021     CLuaUtility::AddToTable(pt_lua_state, "distance_left",  m_sReading.CoveredDistanceLeftWheel );
00022     CLuaUtility::AddToTable(pt_lua_state, "distance_right", m_sReading.CoveredDistanceRightWheel);
00023     CLuaUtility::AddToTable(pt_lua_state, "velocity_left",  m_sReading.VelocityLeftWheel        );
00024     CLuaUtility::AddToTable(pt_lua_state, "velocity_right", m_sReading.VelocityRightWheel       );
00025     CLuaUtility::AddToTable(pt_lua_state, "axis_length",    m_sReading.WheelAxisLength          );
00026     CLuaUtility::CloseRobotStateTable(pt_lua_state);
00027   }
00028 #endif
00029 
00030   /****************************************/
00031   /****************************************/
00032 
00033 #ifdef ARGOS_WITH_LUA
00034   void CCI_DifferentialSteeringSensor::ReadingsToLuaState(lua_State* pt_lua_state) {
00035     lua_getfield  (pt_lua_state, -1, "wheels"                        );
00036     lua_pushnumber(pt_lua_state, m_sReading.CoveredDistanceLeftWheel );
00037     lua_setfield  (pt_lua_state, -2, "distance_left"                 );
00038     lua_pushnumber(pt_lua_state, m_sReading.CoveredDistanceRightWheel);
00039     lua_setfield  (pt_lua_state, -2, "distance_right"                );
00040     lua_pushnumber(pt_lua_state, m_sReading.VelocityLeftWheel        );
00041     lua_setfield  (pt_lua_state, -2, "velocity_left"                 );
00042     lua_pushnumber(pt_lua_state, m_sReading.VelocityRightWheel       );
00043     lua_setfield  (pt_lua_state, -2, "velocity_right"                );
00044     lua_pop       (pt_lua_state, 1                                   );
00045   }
00046 #endif
00047 
00048 
00049   /****************************************/
00050   /****************************************/
00051 
00052   const CCI_DifferentialSteeringSensor::SReading& CCI_DifferentialSteeringSensor::GetReading() const {
00053     return m_sReading;
00054   }
00055 
00056 }
```

---

Generated on 10 Jul 2018 for ARGoS by 
 1.6.1
